# Towards semi-synthetic microbial communities: enhancing soy sauce fermentation properties in *B. subtilis* co-cultures

**DOI:** 10.1186/s12934-019-1149-2

**Published:** 2019-06-03

**Authors:** Rachatida Det-udom, Charlie Gilbert, Long Liu, Cheunjit Prakitchaiwattana, Tom Ellis, Rodrigo Ledesma-Amaro

**Affiliations:** 1 Centre for Synthetic Biology, Imperial College London, London, SW7 2AZ UK; 2 Department of Bioengineering, Imperial College London, London, SW7 2AZ UK; 3 Department of Food Technology, Faculty of Science, Chulalongkorn University, Patumwan, Bangkok, 10330 Thailand; 4 Key Laboratory of Industrial Biotechnology, Ministry of Education, Jiangnan University, Wuxi, 214122 China

**Keywords:** Synthetic microbial communities, Synthetic biology, Metabolic engineering, Soy sauce, Industrial microbiome

## Abstract

**Background:**

Many fermented foods and beverages are produced through the action of complex microbial communities. Synthetic biology approaches offer the ability to genetically engineer these communities to improve the properties of these fermented foods. Soy sauce is a fermented condiment with a vast global market. Engineering members of the microbial communities responsible for soy sauce fermentation may therefore lead to the development of improved products. One important property is the colour of soy sauce, with recent evidence pointing to a consumer preference for more lightly-coloured soy sauce products for particular dishes.

**Results:**

Here we show that a bacterial member of the natural soy sauce fermentation microbial community, *Bacillus*, can be engineered to reduce the ‘browning’ reaction during soy sauce production. We show that two approaches result in ‘de-browning’: engineered consumption of xylose, an important precursor in the browning reaction, and engineered degradation of melanoidins, the major brown pigments in soy sauce. Lastly, we show that these two strategies work synergistically using co-cultures to result in enhanced de-browning.

**Conclusions:**

Our results demonstrate the potential of using synthetic biology and metabolic engineering methods for fine-tuning the process of soy sauce fermentation and indeed for many other natural food and beverage fermentations for improved products.
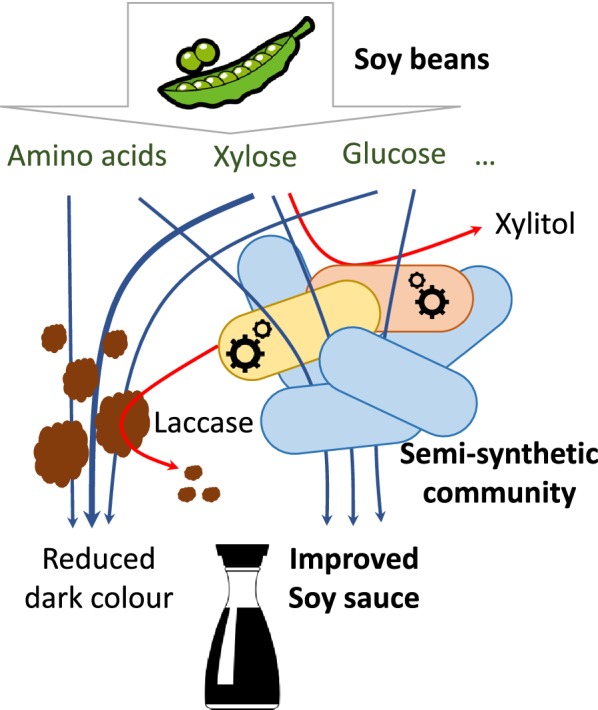

**Electronic supplementary material:**

The online version of this article (10.1186/s12934-019-1149-2) contains supplementary material, which is available to authorized users.

## Background

Fermented foods and beverages are defined as those in which the product is modified by the growth of microbes or communities of microbes. This broad class of foods and beverages is near ubiquitous throughout world. Through fermentation, the resistance to spoiling, flavour, texture, aroma and nutritional content of foods and beverages can all be improved. Many fermented foods and beverages are produced through the action of complex communities of microbes. For instance, sourdough bread is produced through the combined action of lactic acid bacteria, from genera such as *Lactobacillus*, *Pediococcus* and *Leuconostoc*, and of yeasts, such as *Saccharomyces cerevisiae* and *Candida humilis* [1]. Wine is another fermented product consisting of the yeasts *Saccharomyces cerevisiae, Candida* and *Hanseniaspora* species, and bacteria *Oenococcus oeni, Leuconostoc mesenteroides, Pediococcus parvulus,* with complex interactions among them [2].

Recognising the important and beneficial role these microbes and microbial communities can play in food and beverage fermentation, food technologists have sought to harness and control the advantages of fermentation. Fermentation can be controlled by modifying the composition of microbial communities, the microbial culture conditions and the substrate or substrates used. However, this approach is limited by the properties of naturally-occurring microbial communities. Consequently, important characteristics of fermentations, such as the relative growth dynamics, substrate utilisation and metabolic profiles of individual species, cannot easily be controlled.

As a result, there is growing interesting in using genetic engineering approaches to enable rational modification of these characteristics in microbes involved in fermentation. Synthetic biology offers the ideal conceptual framework and genetic tools for achieving this aim by creating semi-synthetic microbial communities, those where one or more engineered organisms are added to a natural community to improve its performance [3]. Metabolic engineering approaches have enabled the production of desirable metabolites, such as vitamins, from microbes and the creation of engineered probiotic therapeutic microbes [4]. In another recent example, an industrial brewing yeast strain was engineered to produce non-native aromatic monoterpenes, imparting hoppy flavours to beer brewed with these strains [5].

**Fig. 1:**
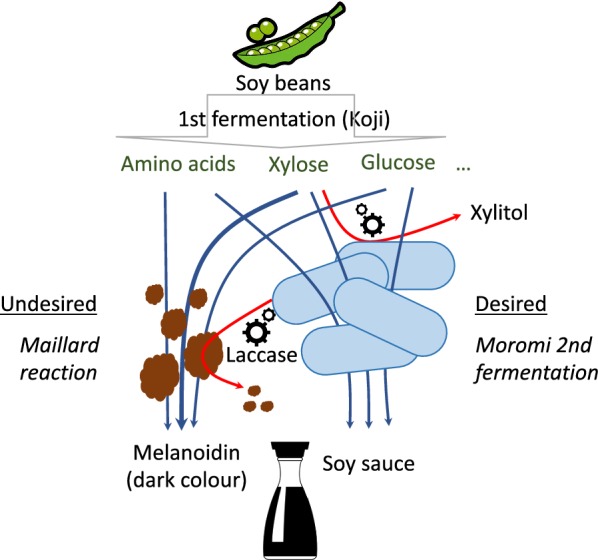
Schematic representation of the soy sauce production. First the Koji fermentation release fermentable sugars and amino acids, which are the substrate for the moromi fermentation and the Maillard reaction. Red arrows indicate the two engineering approaches carried out in this work to reduce the browning of the soy sauce

Soy sauce is one of the world’s most popular condiments with value of $926.2 million USD retail sales and Compound Annual Growth rate (CAGR) during 2017–2021 of 6.20% [6]. Recent studies have shown that there is a growing preference among some consumers for more lightly-coloured soy sauce products [7]. Previous reports have proposed possible solutions for ‘de-browning’ of soy sauce through absorption and filtration, to generate a more lightly-coloured product [8–10]. However, these approaches result in a loss of complexity in the final product compromising sensory attributes of flavors and aromas. Soy sauce production from soy beans consists of two main processes, solid-stage koji fermentation followed by submerged moromi fermentation, each carried out by sequential growth of fungal and bacterial communities throughout the process [11] (Fig. 1). Firstly, microorganisms in the koji fermentation step, particularly members of the *Aspergillus* genus, breakdown complex biomolecules into simpler ones. Here soybean proteins are hydrolysed into small peptides and free amino acids, and sugar polymers, such as gelatinized starch from wheat and soybean, are converted into simple sugars, such as glucose and xylose. In the subsequent moromi brine fermentation, the metabolic products of koji fermentation serve as nutrients for the growth of halophilic bacteria such as lactic acid bacteria (LAB), *Bacillus* species and yeasts, which produce organic acid and/or taste-active compounds [12]. These native, autochthonous microbes produce numerous metabolites giving specific character of soy sauce.

The brown colouration of soy sauce is primarily generated by reactions between reducing sugars and amino acids, commonly known as the Maillard reaction [13]. An important reaction in the production of numerous foods, the Maillard reaction occurs between the carbonyl group of reducing sugars and the amine group of amino acids, creating a highly complex mix of molecules. The efficiency of the Maillard reaction is highly-dependent on the composition of the food stuff, for instance, sugars such as xylose are much more reactive than others such as glucose [14]. Many molecules produced during the Maillard reaction contribute to the flavor and aroma profile of foods. In addition, the Maillard reaction can generate a set of brown pigmented, high molecular weight heterogenous polymers collectively known as melanoidins [15]. During the moromi soy sauce fermentation stage, the Maillard reaction occurs in the fermentation mash, generating the melanoidins which turn soy sauce brown [16]. Reducing the content of these melanoidins in the final soy sauce product might therefore allow the production of a more lightly-coloured soy sauce product.

While various recombinant enzyme-based approaches might be used to prevent the formation of or to degrade melanoidins, these methods would likely substantially increase the costs of soy sauce fermentation. Instead, we set out to genetically engineer a member of the soy sauce fermentation microbial community to enable de-browning of soy sauce. We first identified a candidate chassis organism suitable both for growth under soy sauce fermentation conditions and for de-browning activity. Based on this initial screen, we selected the Gram-positive bacterium *B. subtilis* as our chassis of choice. Using *B. subtilis*, we explored two strategies that could provide soy sauce de-browning activity. Firstly, by engineering catabolism of xylose, the key precursor in the browning process, and secondly, by engineering degradation of melanoidin, the biomolecule responsible for the brown colour of soy sauce.

## Results and discussion

### Identification of candidate chassis of engineering among the natural microbial community members

We first set out to select a suitable chassis organism that we could engineer to carry out soy sauce de-browning. An ideal chassis organism would be both genetically-tractable and exhibit a natural propensity to proliferate in the soy sauce fermentation microbial community. In a previous study, we isolated and characterised various native members of the soy sauce moromi fermentation microbial community [17]. These natural isolates were screened for high salt tolerance (≥ 15% NaCl), for the ability to degrade xylose, a key reducing sugar precursor in the Maillard reaction, and for the ability to break down melanoidins. This process identified a Gram-positive bacterium *Bacillus amyloliquefaciens* strain SSB6 as the isolate exhibiting the highest ‘de-browning’ activity (Additional file 1: Figure S1). When this strain was inoculated into mature moromi, however, it only reduced total browning by 7.85% (unpublished results). This organism thus represents an attractive target for genetic engineering to improve soy sauce de-browning, however it lacks a set of proven synthetic biology tools compared to related bacteria such as *B. subtilis.* Interestingly, *B. subtilis* is also a member of the natural moromi fermentation microbial community. In fact, 18 out of 139 isolates from the moromi microbial community were *B. subtilis* strains, indicating a natural propensity for this bacteria to grow under soy sauce fermentation conditions. As a natural member of the soy sauce moromi microbial community and a model organism for synthetic biology, we set out to engineer *B. subtilis* carry out soy sauce de-browning. In addition, due to being related to *B. amyloliquefaciens* SSB6, we hypothesised that *B. subtilis* might be an ideal chassis to express heterologous enzymes from that organism to improve de-browning.

#### Strategy I: engineering xylose consumption to reduce browning

Xylose is a highly-reactive sugar in the Maillard reaction, which produces the melanoidins responsible for soy sauce browning. In Thai soy sauce moromi, the most abundant sugars are xylose and glucose, which are in a ratio of 1:10 respectively [18, 19], xylose being the major contributor to the Maillard reaction. Therefore, to reduce the degree of browning occurring during moromi fermentation, we set out to engineer the degradation of xylose in *B. subtilis*. Although *B. subtilis* possess a native pathway for xylose metabolism, via conversion to xylulose and the pentose phosphate pathway, we set out to engineer a novel xylose-degradation pathway in *B. subtilis* which would result in incomplete degradation of xylose to xylitol. Specifically, we set out to engineer expression of xylose reductase (XR) in *B. subtilis*. XR catalyses the conversion of xylose to xylitol, a high value sugar alcohol used extensively in food products. Xylitol itself is an anticariogenic and antiketogenic molecule and a sweetener. Importantly, xylitol lacks a carbonyl group and hence is unable to react via the Maillard reaction. Therefore, we hypothesised that conversion of xylose to xylitol might reduce browning.

**Fig. 2:**
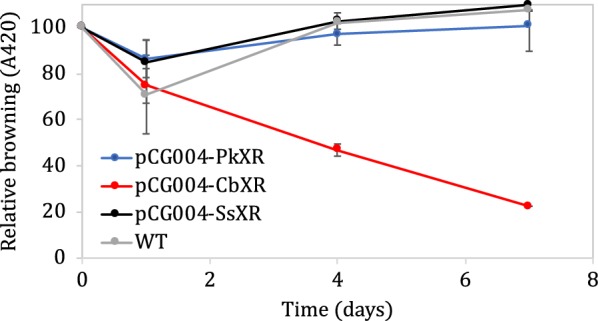
Relative browning compare to initial conditions (100%) in wild type and strains engineered to express xylose reductases cultured in inducing M9 media. Samples prepared in duplicate, error bars represent ± SD

We engineered *B. subtilis* to express fungal XRs from *Pichia kudriavzevii* (pCG004-amyQ SP-PkXR), *Candida boidinii* (pCG004-amyQ SP-CbXR) and *Scheffersomyces stipites* (pCG004-amyQ SP-SsXR). All XR genes were cloned under the control of a plasmid-borne, IPTG-inducible promoter (P_grac_). Strains expressing XRs were first screened for the ability to degrade xylose and produce xylitol as measured by HPLC (Additional file 1: Figure S2). While *B. subtilis* strains expressing pCG004-amyQ SP-PkXR and pCG004-amyQ SP-SsXR resulted in no appreciable increase in xylitol or decrease in xylose, the strain expressing pCG004-amyQ SP-CbXR exhibited a sharp production of xylitol (2 g/L), indicating expression of functional XR (Additional file 1: Figure S2). To test whether expression of XR lead to a reduction of browning through the Maillard reaction, culture supernatants from cells grown in xylose-containing medium were collected, boiled to initiate Maillard reaction and then the A_420_ measured to determine the degree of browning (Fig. 2). While expression of pCG004-amyQ SP-PkXR and pCG004-amyQ SP-SsXR led to no substantial decrease in the browning caused by the Maillard reaction, pCG004-amyQ SP-CbXR expression resulted in a clear decrease in browning of 80% compared with the non-engineered strain.

**Fig. 3:**
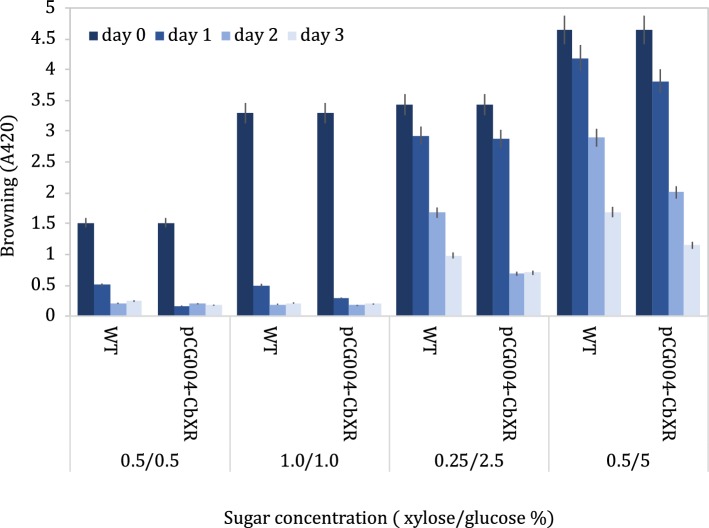
Browning of wild-type (WT) and strains engineered to express xylose reductases in mixed sugars at various ratios. X-axis shown the amount and ratio of xylose to glucose, respectively (% xylose/% glucose). Samples prepared in duplicate, error bars represent ± SD

However, in reality, soy sauce fermentations consist of a complex mixture of carbohydrates, with glucose concentrations likely to be in excess of xylose concentrations. We therefore tested whether the engineered *B. subtilis* pCG004-amyQ SP-CbXR strain could reduce browning in conditions more closely matching soy sauce fermentation. Cultures were grown in medium with glucose and xylose sugars at a range of different concentrations, including a 1:10 xylose-to-glucose ratio, which is typical of soy sauce fermentations. Spent media were boiled after various time points to initiate the Maillard reaction and produce melanoidins and the absorbance at 420 nm (A_420_) of resultant samples was measured to determine the degree of browning (Fig. 3). At lower sugar concentrations, all sugars were rapidly consumed, resulting in the production of less melanoidin and hence less browning. At higher sugar concentrations, *B. subtilis* pCG004-amyQ SP-CbXR spent media exhibited a marked reduction in browning compared to the wild-type control. Therefore, *B. subtilis* pCG004-amyQ SP-CbXR was able to consume xylose in mixed sugar media, resulting in decreased browning of the medium. After 3 days, the browning reduction in the engineered strain were 80.0% and 56.8% from 0.25/2.5 and 0.5/5.0 xylose to glucose respectively while in the wild type the reduction was only 61.1% and 27.7% for the respective conditions.

#### Strategy II: engineering enzymatic degradation of melanoidin pigments

In a second approach, we hypothesised that de-browning of soy sauce could also be achieved by engineering enzymatic degradation of the species responsible for the brown colour, namely melanoidins. Laccases are a group of copper-containing oxidase enzymes with broad substrate specificities and a number of potential uses, such as degradation of xenobiotics and dyes from industrial wastewater. In fact, laccases have been previously shown to degrade melanoidin [20]. We therefore set out to engineer *B. subtilis* to secrete laccase enzymes to enable melanoidin degradation during soy sauce fermentation.

**Fig. 4:**
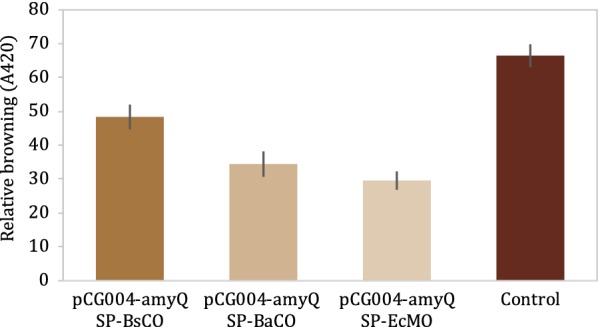
Relative browning compared to initial conditions of cell-free supernatant incubated with 1% synthetic melanoidin of wild-type (WT) and laccase expressing strains. Samples prepared in duplicate, error bars represent ± SD

*Bacillus subtilis* strains were engineered to express laccases from *B. subtilis cot*A, *B. amyloliquefaciens* copper oxidase, and *Escherichia coli* multicopper oxidase genes, each fused to the highly-efficient *B. amyloliquefaciens* AmyQ signal peptide to direct protein secretion. Secreted proteins were tested for browning reduction ability using synthetic melanoidin. In all conditions, the A_420_ decreased over the course of 7 days, including in the wild-type *B. subtilis* negative control sample, which may be due to background expression of native *B. subtilis* laccase enzymes. However, the three engineered laccase-expressing strains directed increased degradation of melanoidins compared to the control, with pCG004-amyQ SP-EcMO resulting in the greatest degree of de-browning (70.4% reduction) (Fig. 4). Therefore, taking advantage of the ability of *B. subtilis* to secrete heterologous proteins, we were able to engineer melanoidin-degrading strains.

### Co-culturing engineered xylose-consuming and melanoidin-degrading strains to enhance soy sauce de-browning

**Fig. 5:**
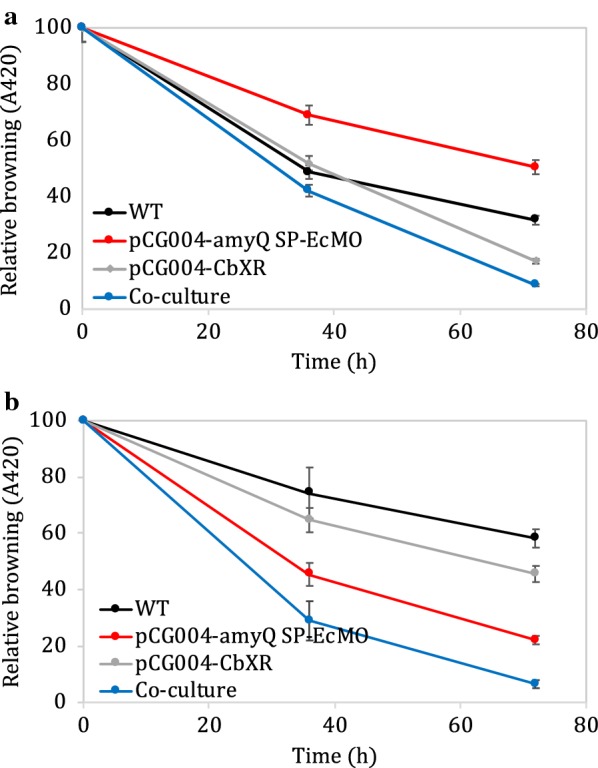
Relative browning compared to initial conditions in the co-culture experiment. **a** Analysis of the effect of reducing the sugars available in the media. Boiled, cell-free supernatant of either wild-type (WT), *B. subtilis* expressing pCG004-CbXR, pCG004-amyQ SP-EcMO or co-cultured of *B. subtilis* expressing pCG004-CbXR and pCG004-amyQ SP-EcMO clones in media containing 5% glucose and 0.5% xylose at different cultivation time; **b** analysis of the melanoidin degrading activity in the culture media after growth. 2-days incubation mixture of boiled and reserved unboiled supernatant of either wild-type (WT), *B. subtilis* expressing pCG004-CbXR, pCG004-amyQ SP-EcMO or co-cultured of *B. subtilis* expressing pCG004-CbXR and pCG004-amyQ SP-EcMO clones in media containing 5% glucose and 0.5% xylose at different cultivation time. Samples prepared in duplicate, error bars represent ± SD

We hypothesised that combining both strategies for soy sauce de-browning might exhibit a synergistic effect and result in enhanced de-browning. To test this, we prepared mono-cultures and a co-culture of the best-performing strains from each approach: *B. subtilis* pCG004-CbXR and *B. subtilis* pCG004-amyQ SP-EcMO. Culture supernatants were harvested from these cultures at various time points and boiled to induce browning through the Maillard reaction. Firstly, de-browning activity due to xylose degradation was assessed by monitoring the A_420_ of the resultant samples (Fig. 5a). As expected, both *B. subtilis* pCG004-CbXR and co-culture samples exhibited reduced browning compared to the wild-type and *B. subtilis* pCG004-amyQ SP-EcMO samples. Interestingly, the *B. subtilis* pCG004-amyQ SP-EcMO sample showed increased browning compared to wild-type, which may be caused by a slightly slower growth rate of this strain compared to wild-type. Slower growth might result in decreased consumption of reducing sugars which are substrates for the Maillard reaction. Secondly, to measure the effect of laccase expression on de-browning, we next added unboiled supernatant samples to the boiled ones (in which the Maillard reaction had occurred) and incubated them for 2 days at 37 °C, after which the A_420_ was measured (Fig. 5b). As expected, compared to the wild-type, both the *B. subtilis* pCG004-amyQ SP-EcMO and co-culture samples exhibited decreased browning. The additive effects of xylose consumption and melanoidin degradation meant that boiled and treated co-culture samples exhibited the greatest degree of de-browning.

## Conclusions

In this work, we used synthetic biology to engineer *B. subtilis*, an organism found in moromi, in order to provide it with enhanced de-browning properties that are beneficial during soy sauce fermentation. We here generated two strains capable of reducing the production of melanoidins during the Maillard reaction, the major contributors to the brown colour of the sauce. *B. subtilis* pCG004-CbXR expressed a xylose reductase, an enzyme that converts xylose (the sugar that contributes the most to the Maillard reaction) into xylitol (a desired compound that contribute to the sensory quality of foods). This strain showed a significant reduction in brown colour. In addition, *B. subtilis* pCG004-amyQ SP-EcMO, secreted a laccase, an enzyme that degrades the melanoidins. This strain also showed a reduction of browning after the Maillard reaction. Finally, we proved the additive effect of these two strains when grown together as a co-culture: the co-culture showed more reduced browning than the individual strains.

In future approaches the same strain can be engineered to express both activities and similar modifications can be engineered in other natural isolates of moromi. In addition, this proof of concept, carried out in synthetic media and induced Maillard reaction, paves the way to study semi-synthetic communities in soy sauce industrial fermentation to evaluate its de-browning effect in long-term processes. For this, the constitutive expression of the selected enzymes, integrated in the genome would be preferred in order to increase stability. Although differences in the absolute de-browning values shown here are expected in the soy sauce fermentation due to the different composition of nutrients, the strains should still contribute to de-browning by their enhanced capacity to convert xylose and degrade melanoidins. Although significant changes in the flavour of soy sauce would not be expected when the strains have been isolated from moromi, this is something that needs to be analysed.

The concept of a semi-synthetic microbial community, and the creation of engineered strains that could benefit natural microbial communities by adding novel capabilities, can be applied to any microbial fermentation. The process can start by identifying the members of the community (for example by metagenomics) and characterising the environment they grow in, including the available substrates. Then key isolates (or their close relatives) can be engineered using synthetic biology to provide the desired properties for the final product. Finally, the improved microbes can be introduced at different proportions mixed with the natural inoculum in order to identify their peak performance and contribution to the product. This whole process will be refined in the near future due to advances in our understanding of microbial community behaviours and through our abilities to engineer biology in more precise manner.

## Materials and methods

### Strains and plasmids

Bacterial strains and plasmids used in this study were shown in Additional file 1: Tables S1 and S2, respectively. Both bacteria were cultured in LB or M9 medium with an aeration condition at 37 °C. antibiotics were used as selective marker at appropriate concentrations of 34 µg/mL chloramphenicol or 100 μg/mL ampicillin for *E. coli* Turbo and 5 µg/mL chloramphenicol for *B. subtilis*, depending on plasmid type.

### Construction of plasmid

All plasmids used in this study were transformed into *E. coli* turbo (NEB, USA) for amplification and sequence-verified by Sanger sequencing (Sourcebioscience, United Kingdom). The verified plasmids were then clone into *B. subtilis* host cell for protein expression and secretion in case of laccase.

For xylose reductase approach, BsaI/BsmbI golden gate assemble system was used to construct pCG004-CbXR, pCG004-PkXR and pCG004-SsXR from optimised oligonucleotides of *C. boidinii* aldose reductase (CbXR), *P. kudriavzevii* NADPH-dependent d-xylose reductase (PkXR) and *Scheffersomyces stipitis* XylI (SsXR), respectively (Additional file 1: Table S2). Codon optimization tool (IDTDNA, USA) was used in oligonucleotides from *E. coli* to create the optimized-Bacillus coding sequences.

Similarly, oxidoreductase-related genes from *Bacillus* species and *E. coli* were designed by introduction of the upstream and downstream restriction enzyme sites (Additional file 1: Table S3).

For oxidoreductase-constructed plasmid, pYTK001 was used as primary backbone and pCG004 as a secondary. The first assemble with pYTK001 was done with BsmBI restriction enzyme. Oligonucleotide of *B. subtilis* subsp. subtilis str. 168 copper oxidase (BsCO), *B. amyloliquefaciens* DSM 7 copper oxidase (BaCO) and *E. coli* str. K-12 substr. MG1655 multicopper oxidase (EcMO) were introduced into the first backbone. Verified pYTK001-BsCO, pYTK001-BaCO and pYTK001-EcMO were then ligated with amyQ SP and pCG004 using BsaI restriction enzyme, resulting in pCG004-amyQ SP-BsCO, pCG004-amyQ SP-BaCO and pCG004-amyQ SP-EcMO.

### Protein expression

A single colony of *B. subtilis* carrying either xylose reductase or oxidoreductase encoding genes was inoculated into LB medium and incubated at 37 °C. After 16 h, cell suspension was diluted by M9 media until an absorbance at 600 nm was reach 0.1. Protein expression was induced with 1 mM IPTG.

### Browning reduction

#### Xylose reductase scheme

IPTG-inducing media of M9 containing 3% xylose or glucose was inoculated with pCG004-CbXR, pCG004-PkXR or pCG004-SsXR *Bacillus* and incubated at 37 °C for 7 days with aeration. Cell-free supernatant collected at day 4 and 7 was divided into 2 parts, the first was subjected to analysis for available xylose and xylitol by HPLC. The second part was subjected to browning induction before determination of colour took place.

The effect of xylose reductase expression toward browning mitigation was further investigated in mixed sugar species of glucose and xylose. Various sugar concentrations including equal proportions of 0.5% and 1%, and 1:10 ratio of xylose to glucose (0.25:2.5 and 0.5:5.0%) were applied with same cultivation condition as previous experiment for 3 days. Cell-free supernatant was collected every 24 h for browning induction and determination.

Browning induction was done by boiling those collected supernatants at 100 °C for 4 h. to induce Maillard reaction. Browning of boiled supernatant was then determined by spectrophotometer at absorbance of 420 [13].

#### Oxidoreductase scheme

Clones of pCG004-amyQ SP-BsCO, pCG004-amyQ SP-BaCO and pCG004-amyQ SP-EcMO were cultured in IPTG-inducing M9 media for 7 days at 37 °C, aeration condition. Any cell debris was removed from liquid part and supernatant was collected for determination of browning reduction via secreted oxidoreductase protein.

Verification of an activity of *Bacillus* secreting proteins in browning reduction was done with synthetic melanoidin unless stated otherwise. The synthesis condition was modified from Murata, Terasawa and Homma [21] as the solution containing 10% of xylose and soy peptone. Synthesized melanoidin was then added into cell-free supernatant to the final concentration of 1% and incubated at 37 °C with aeration for 2 days. Browning was observed spectrophotometry as stated in 2.5.1

### Co-culture of engineered strains

Culture of potential xylose reductase and oxidoreductase clones was inoculated into IPTG-inducing M9 media containing 5% glucose and 0.5% xylose for 24, 48 and 72 h. Each interval, 2 mL supernatant was collected and separated into 2 tubes. The first tube followed an induction of the Maillard reaction by boiling at 100 °C for 4 h. Then, browning determination took place in order to evaluate xylose reduction. The second tube was used to verify melanoidin degradation by mean of oxidoreductase activity. For this, we followed the protocol in 2.5.2 but using boiled supernatant from the first tube instead of synthetic melanoidin.

## Additional file

**Additional file 1: Table S1.** Bacterial strains used in this study. **Table S2.** Plasmids used in this study. **Table S3.** DNA sequences of the genes used in this study. **Figure S1.** De-browning effect of *B. amyloliquefaciens strain SSB6*. **Figure S2.** Xylose utilization and xylitol production of engineered strains.

## Data Availability

All data generated or analysed during this study are included in this published article and its additional files.
