# Supplementary material for: Towards semi-synthetic microbial communities: enhancing soy sauce fermentation properties in B. subtilis co-cultures
Source: Microb Cell Fact. 2019 Jun 3;18:101. doi: 10.1186/s12934-019-1149-2 (PMC6547557; doi:10.1186/s12934-019-1149-2)
Supplement: Supplementary file 1 — Additional file 1: Table S1. Bacterial strains used in this study. Table S2. Plasmids used in this study. Table S3. DNA sequences of the genes used in this study. Figure S1. De-browning effect of B. amyloliquefaciens strain SSB6. Figure S2. Xylose utilization and xylitol production of engineered strains. [file 12934_2019_1149_MOESM1_ESM.docx]

**Towards semi-synthetic microbial communities: enhancing soy sauce fermentation properties in *B. subtilis* co-cultures**

Rachatida Det-udom, Charlie Gilbert, Cheunjit Prakitchaiwattana,

Tom Ellis and Rodrigo Ledesma-Amaro

**Additional Figures and Tables**

**Additional table S1**: Bacterial strains used in this study.

| Name | Description | Source |
| --- | --- | --- |
| Bacillus subtilis WB800N | trpC2 nprE aprE epr bpr mpr::ble nprB::bsrΔvpr wprA::hyg cm::neo; NeoR | MoBiTec |
| Escherichia coli Turbo | F' *proA^+^ B^+^ lacI^q^ ∆lacZM15 / fhuA2 ∆(lac-proAB) glnV galK16 galE15 R(zgb210::Tn10)Tet^S^ endA1 thi-1 ∆(hsdS-mcrB)5* | NEB |

**Additional table S2**: Plasmids used in this study.

| Name | Description | Source |
| --- | --- | --- |
| pYTK001 | An entry vector taken from the yeast tool kit. | Lee *et al*, 2015 |
| pCG004 | ORF part entry vector derived from *B. subtilis-E.coli* shuttle vector consists of GFP-expression cassette and IPTG-inducible protein expression promoter ORF part assembly entry vector. | Gilbert *et al*, 2017 |
| pCG-AmyQ SP-B | an ORF-part containig vector with the signal peptide from *B. amyloliquefaciens* AmyQ signal peptide | Gilbert *et al*, 2017 |
| pYTK001-BsCO | An ORF part-containing vector harbouring the signal peptide from *B. subtilis* cotA | This work |
| pYTK001-BaCO | An ORF part-containing vector harbouring the signal peptide from *B. amyloliquefaciens* copper oxidase | This work |
| pYTK001-EcMO | An ORF part-containing vector harbouring the signal peptide from *E. coli* multicopper oxidase | This work |
| pCG004-amyQ SP-BsCO | An ORF part-containing vector harbouring the signal peptide from pCG-AmyQ SP-B and *B. subtilis cot*A | This work |
| pCG004-amyQ SP-BaCO | An ORF part-containing vector harbouring the signal peptide from pCG-AmyQ SP-B and *B. amyloliquefaciens* copper oxidase | This work |
| pCG004-amyQ SP-EcMO | An ORF part-containing vector harbouring the signal peptide from pCG-AmyQ SP-B and *E. coli* multicopper oxidase | This work |
| pCG004-CbXR | An ORF part-containing vector harbouring the signal peptide from *Pichia kudriavzevii* NADPH-dependent D-xylose reductase | This work |
| pCG004-PkXR | An ORF part-containing vector harbouring the signal peptide from *Candida boidinii*aldose reductase | This work |
| pCG004-SsXR | An ORF part-containing vector harbouring the signal peptide from *Scheffersomyces stipitis* XylI | This work |

**Additional table S3**: Amino acid sequences of protein parts used in this study.

| Names | Sequences* | Descriptions |
| --- | --- | --- |
| amyQ SP | ggtctcaggatATGATTCAAAAACGAAAGCGGACAGTTTCGTTCAGACTTGTGCTTATGTGCACGCTGTTATTTGTCAGTTTGCCGATTACAAAAACATCAGCCGGaagccgagacc | amyQ SP signal peptide |
| BsCO | tacgcgtctcatcggggtctcaaagcATGACACTTGAAAAATTTGTGGATGCTCTCCCAATCCCAGATACACTAAAGCCAGTACAGCAATCAAAAGAAAAAACATACTACGAAGTCACCATGGAGGAATGCACTCATCAGCTCCATCGCGATCTCCCTCCAACCCGCCTGTGGGGCTACAACGGCTTATTTCCGGGACCGACCATTGAGGTTAAAAGAAATGAAAACGTATATGTAAAATGGATGAATAACCTTCCTTCCACGCATTTCCTTCCGATTGATCACACCATTCATCACAGTGACAGCCAGCATGAAGAGCCCGAGGTAAAGACTGTTGTTCATTTACACGGCGGCGTCACGCCAGATGATAGTGACGGGTATCCGGAGGCTTGGTTTTCCAAAGACTTTGAACAAACAGGACCTTATTTCAAAAGAGAGGTTTATCATTATCCAAACCAGCAGCGCGGGGCTATATTGTGGTATCACGATCACGCCATGGCGCTCACCAGGCTAAATGTCTATGCCGGACTTGTCGGTGCATATATCATTCATGACCCAAAGGAAAAACGCTTAAAACTGCCTTCAGACGAATACGATGTGCCGCTTCTTATCACAGACCGCACGATCAATGAGGATGGTTCTTTGTTTTATCCGAGCGCACCGGAAAACCCTTCTCCGTCACTGCCTAATCCTTCAATCGTTCCGGCTTTTTGCGGAGAAACCATACTCGTCAACGGGAAGGTATGGCCATACTTGGAAGTCGAGCCAAGGAAATACCGATTCCGTGTCATCAACGCCTCCAATACAAGAACCTATAACCTGTCACTCGATAATGGCGGAGATTTTATTCAGATTGGTTCAGATGGAGGGCTCCTGCCGCGATCTGTTAAACTGAATTCTTTCAGCCTTGCGCCTGCTGAACGTTACGATATCATCATTGACTTCACAGCATATGAAGGAGAATCGATCATTTTGGCAAACAGCGCGGGCTGCGGCGGTGACGTCAATCCTGAAACAGATGCGAATATCATGCAATTCAGAGTCACAAAACCATTGGCACAAAAAGACGAAAGCAGAAAGCCGAAGTACCTCGCCTCATACCCTTCGGTACAGCATGAAAGAATACAAAACATCAGAACGTTAAAACTGGCAGGCACCCAGGACGAATACGGCAGACCCGTCCTTCTGCTTAATAACAAACGCTGGCACGATCCCGTCACAGAAACACCAAAAGTCGGCACAACTGAAATATGGTCCATTATCAACCCGACACGCGGAACACATCCGATCCACCTGCATCTAGTCTCCTTCCGTGTATTAGACCGGCGGCCGTTTGATATCGCCCGTTATCAAGAAAGCGGGGAATTGTCCTATACCGGTCCGGCTGTCCCGCCGCCGCCAAGTGAAAAGGGCTGGAAAGACACCATTCAAGCGCATGCAGGTGAAGTCCTGAGAATCGCGGCGACATTCGGTCCGTACAGCGGACGATACGTATGGCATTGCCATATTCTAGAGCATGAAGACTATGACATGATGAGATCGATGGATATAACTGATCCCCATAAATAAgttctgagaccgacctgagacgatgc | Full length *Bacillus subtilis* subsp. *subtilis* str. 168 copper oxidase gene |
| BaCO | tgaccgtctcatcggggtctcaaagcATGGCACTTGAAAAATTTGCAGATGAACTGCCGATTATCGAAACACTGAAGCCGCAGAAGACATCAAACGGCAGCACGTATTATGAAGTCACGATGAAGGAATGCTTTCACAAGCTGCACCGTGATCTCCCGCCGACCCGGCTGTGGGGCTATAACGGTTTGTTTCCCGGCCCGACGATCGACGTGAACCAAGATGAGAACGTCTATATTAAATGGATGAATGACCTGCCGGATAAGCATTTTCTCCCTGTGGACCATACCATTCACCATTCAGAGGGCGGCCATCAGGAACCCGACGTCAAAACCGTCGTCCATTTACACGGAGGAGCAACGCCGCCGGACAGCGACGGCTATCCGGAAGCCTGGTTCACACGGGATTTCAAGGAGAAGGGGCCTTATTTTGAAAAAGAGGTATACCACTATCCAAACAAACAGCGCGGGGCGCTATTATGGTATCACGACCACGCCATGGCAATTACGAGGCTCAATGTGTACGCCGGGCTTGCCGGCATGTATATCATCCGCGAGCGAAAAGAAAAGCAGCTGAAGCTTCCCGCCGGAGAATACGACGTACCGCTTATGATTATGGACCGCACGTTAAATGACGACGGTTCCTTGTTTTATCCGAGCGGGCCCGATAATCCTTCCGAAACGCTGCCGAATCCTTCAATCGTTCCATTCCTTTGCGGAAATACCATTCTCGTCAACGGCAAAGCGTGGCCGTATATGGAAGTCGAACCGCGGACATATCGTTTCCGTATCCTTAACGCCTCAAATACGAGAACATTTTCCCTCTCGCTCAATAATGGCGGCCGGTTTATTCAAATCGGTTCTGACGGCGGACTGCTCCCCCGTTCTGTCAAGACACAGTCCATCAGCTTAGCCCCGGCTGAGCGGTATGATGTGCTCATTGATTTCTCCGCTTTTGACGGAGAACATATTATTTTAACGAACGGCACCGGCTGCGGGGGCGACGTCAATCCGGATACCGACGCCAATGTGATGCAATTCCGCGTCACAAAACCGCTGAAGGGAGAAGACACCAGCCGGAAGCCTAAATATCTGTCAGCCATGCCTGATATGACATCAAAAAGAATACACAATATCAGGACGCTTAAACTCACAAACACGCAAGACAAATACGGCCGGCCGGTTTTAACACTCAATAACAAGCGCTGGCATGATCCCGTGACAGAAGCGCCGCGGCTCGGCTCAACGGAAATCTGGTCGATTATCAACCCGACGCGGGGAACCCATCCGATACACCTGCACTTGGTTTCCTTCCAAGTCCTTGACCGGCGTCCTTTTGACTTAGAACGTTATAACAAATTCGGCGACATTGTGTATACAGGCCCCGCCGTCCCGCCGCCTCCAAGTGAAAAAGGCTGGAAAGACACCGTGCAGGCGCACTCCGGAGAAGTCATCAGAATCGCGGCGACATTCGCGCCTTACAGCGGACGGTACGTATGGCATTGTCATATTTTAGAACATGAAGATTATGACATGATGAGGCCGATGGACGTCACAGAAAAGCAGTAAgttctgagaccgacctgagacgatgc | Full length *Bacillus amyloliquefaciens* DSM 7 copper oxidase gene |

*Uppercases illustrated amino acid sequence of target genes, while lowercases were overhangs for restriction enzymes

**Additional table S3**: Amino acid sequences of protein parts used in this study. (continue)

| Names | Sequences* | Descriptions |
| --- | --- | --- |
| EcMO | tgaccgtctcatcggggtctcaaagcATGCAACGCCGTGACTTTCTTAAATACTCAGTTGCCCTGGGGGTGGCGTCTGCCTTGCCTCTGTGGTCCCGGGCTGTTTTCGCCGCTGAGCGTCCAACCTTGCCAATTCCTGATCTCCTTACAACTGATGCGCGTAATCGGATCCAACTGACAATCGGCGCAGGCCAGTCTACGTTTGGTGGTAAAACGGCAACGACGTGGGGATATAATGGCAACCTCTTAGGACCGGCTGTGAAATTACAAAGAGGGAAGGCCGTTACTGTGGATATTTACAATCAACTTACCGAGGAAACAACTCTTCACTGGCATGGTCTGGAAGTGCCAGGGGAAGTTGATGGGGGACCTCAAGGTATAATTCCGCCTGGGGGGAAACGTAGTGTGACGTTGAACGTGGACCAGCCGGCCGCTACTTGCTGGTTCCACCCGCACCAACATGGTAAAACTGGGCGCCAGGTGGCAATGGGGCTCGCTGGACTGGTTGTGATTGAGGATGATGAGATTCTCAAGCTCATGTTACCTAAACAGTGGGGCATCGATGACGTACCTGTCATTGTGCAAGATAAAAAATTCAGTGCTGACGGACAAATCGATTACCAGTTAGATGTTATGACAGCAGCGGTTGGTTGGTTTGGGGATACACTTCTGACAAACGGTGCTATCTATCCGCAGCACGCAGCTCCTCGGGGCTGGCTTCGTTTGCGTCTTTTGAATGGATGCAATGCCCGGAGCCTCAATTTCGCGACCAGCGATAATCGTCCATTATACGTCATCGCCTCAGATGGGGGATTATTGCCGGAACCTGTGAAGGTCAGTGAACTTCCTGTGCTGATGGGTGAACGCTTTGAGGTGCTCGTTGAGGTGAATGATAATAAACCGTTTGACCTTGTCACGTTGCCAGTATCACAGATGGGCATGGCCATTGCTCCATTTGACAAGCCTCACCCAGTAATGCGCATACAACCTATCGCTATATCAGCTAGTGGAGCACTGCCTGATACTTTATCTTCCCTTCCTGCTCTGCCTTCACTTGAGGGACTTACTGTAAGAAAATTGCAACTCTCCATGGACCCGATGCTCGACATGATGGGAATGCAGATGCTGATGGAAAAATACGGcGACCAGGCTATGGCAGGCATGGACCACTCACAAATGATGGGTCATATGGGCCACGGTAACATGAACCATATGAACCATGGGGGGAAATTCGACTTCCACCATGCAAATAAAATAAACGGGCAAGCATTTGACATGAACAAACCGATGTTTGCCGCGGCCAAGGGTCAATATGAGCGGTGGGTTATCTCCGGTGTCGGTGACATGATGTTGCATCCTTTTCACATCCACGGTACGCAATTCAGAATATTATCAGAAAATGGGAAACCACCGGCAGCGCACCGGGCTGGTTGGAAAGATACTGTTAAAGTGGAGGGGAATGTTTCAGAGGTTCTTGTTAAATTTAACCATGACGCGCCTAAAGAGCACGCGTATATGGCCCACTGCCACTTGCTGGAACACGAGGACACTGGCATGATGCTCGGCTTCACGGTGTAAgttctgagaccgacctgagacgatgc | Full length *Escherichia coli* str. K-12 substr. MG1655 multicopper oxidase gene, optimized |
| CbXR | tcggggtctcaggatATGAGTAGTCCGCTGTTAACACTTAATAACGGGTTAAAGATGCCGCAAATCGGCTTTGGTTGTTGGAAGGTAGACAACGCAACCTGTGCGGAGACAATCTATGAGGCTATCAAGGTGGGATATCGCCTGTTCGACGGCGCAATGGATTACGGCAACGAGAAAGAAGTAGGCGAGGGAGTGAATAAGGCGATAAAGGACGGCTTGGTTAAGCGGGAGGAATTATTCATCGTGAGCAAGTTGTGGAATAATTTTCATCATCCTGATTCTGTAAAGTTAGCAATTAAAAAGGTACTGTCAGATCTGAATTTGGAGTACATTGACCTGTTCTACATGCACTTTCCAATTGCACAAAAATTCGTGCCGATTGAAAAAAAATACCCACCGAACTTCTACTGCGGCGACGGTGATAAATGGTCATTTGAGGATGTCCCATTATTGACGACGTGGCGGGCAATGGAGGAGCTTGTTGAAGAGGGACTTGTCAAATCTATAGGAATATCCAATTTTGTTGGGGCTCTGATCCAGGACTTATTACGTGGATGCAAAATACGGCCAGCCGTATTGGAAATCGAGCACCATCCATACCTGGTGCAGCCTCGTCTGATAGAATATGCCAAGACCGAAGGTATCCATGTGACTGCCTATTCATCTTTCGGGCCTCAGTCATTTGTCGAACTCGATCATCCTAAGGTTAAGGATTGTACGACACTGTTCAAGCATGAAACCATAACTTCAATTGCCTCTGCTCACGACGTACCACCAGCCAAAGTGCTCCTCCGGTGGGCAACCCAGCGCGGTTTAGCAGTGATTCCGAAGAGTAACAAAAAAGAACGCCTTTTGGGCAACTTGAAGATTAATGACTTTGATCTTACGGAAGCCGAGTTAGAAAAAATAGAGGCTCTCGACATAGGTTTGCGGTTCAATGACCCTTGGACGTGGGGCTACAATATCCCAACTTTTATATAAgttctgagaccgacc | Full length *Candida boidinii*aldose reductase gene, optimized |

*Uppercases illustrated amino acid sequence of target genes, while lowercases were overhangs for restriction enzymes

**Additional table S3**: Amino acid sequences of protein parts used in this study. (continue)

| Names | Sequences* | Descriptions |
| --- | --- | --- |
| PkXR | tcggggtctcaggatATGAGTCAAGTGTACGTAACCCTCAACAATGGGATCAAGATACCTCAGGTAGGCTTTGGTTGCTGGAAGTTAGTCAATGAAGTTGCTGCCGACCAGATCTACGAAGCAATCAAGATAGGGTACAGATTATTCGATGGAGCACAAGATTATGGAAATGAGAAAGAGATAGGCCAGGGCATTAAGCGTGCAATCAAGGAAGGGATAGTGAAAAGAGAGGATCTTGTTGTCGTCAGTAAACTGTGGAACAGTTTCCACGACCCAAAAAATGTTGAGGTAGCCATCAATAAGGTACTCTCAGACTTAGATTTGGACTATCTTGACATATTTTATATCCATTTTCCAATAGCGCAGAAGTTTGTTCCTATCGAAAAGAAATATCCGCCAGGCTTTTACTGTGGGGAAAACGGCTGGGAATTCGAAGACGTTCCACTTTCCGTAACCTGGAAGGCCATGGAAAACCTCGTGGACCAGGGCAAAGTGAAATCAATCGGCATCAGTAATTGTAATGGGGCGTTGGTTCAGGATCTTTTACGGTCCGCGCGGATTAAACCGCAGCTTTTGCAAATAGAGCATCACCCTTACCTCGTGCAACCACGGCTCGTGAAGTACGCTCAAGACAATGGGATTCATGTCGTAGCGTATTCCTCATTTGGACCGCAATCATTTCTCGAACTCGACCATCCGAAGGCCAAGGACACAGTATCCCTGTTTGAACACGATACGATAAAGGAGATCGCAGCAAAGCATAACGTGTCAACTTCCAAGGTGTTGTTACGGTGGGCGACTCAAAATGGAGTGTTGGTGATTCCTAAGTCCAATCGTAAGGAAAGATTACTCGAGAACTTTAGCGTAAATGACTTCCAACTGGATGAAGAGGACATGAACAAGATAACGGGCCTCGATATGAATTTAAGATTCAACGATCCATGGACGTGGGGTGCAGAGATTCCTACATTTGTATAAgttctgagaccgacc | Full length *Pichia kudriavzevii NADPH-dependent* D-xylose reductase gene, optimized |
| SsXR | tcggggtctcaggatATGAGCTTCAAATTAGCGAGTGGCAAATCAATGCCAAAGGTAGGGTTCGGACTGTGGAAAGTCCCGAGAGATAAAACTGCCGATACTGTATACGGAGCAATTAAAAACGGCTACCGCTTGTTTGACGGTGCGTTCGATTATCAGAATGAGCGCGAAGCGGGCGAAGGAATTCGCCGGGCTATCAAGGATGGATTAGTCAAGCGTGAAGATATCTTTATCACTACAAAGTTGTGGAACACTTTCCATAGTAAAGAACACGCACTCCAGATTGCTAAGGAACAGAATGAATGGTGGGGGTTAGACTACATTGACCTGTACCTTATCCATTTCCCTATTCCAATGCAATATATACCGATAAGTGAAAAGGAATGGGCCGGTTGGACTAACGCTACGGACTCAGGCCCTAACCCACTGGCAAAGATTCCAACTCGGGAGACATGGGAAGCTCTGGAGGAACTGGTAGATACTGGGATAGCAAAGTCCATCGGGGTCAGCAACTTTACCGCGCAAAATATATACGATGTACAAACTTATAACAAGCACCCAATTTCTGCACTTCAAATTGAGCATCACCCATATTTAGTACAGCCACAATTAACCCAGTTAGCCAAAGACAACAATATACAAGTAACTGCTTATTCTTCATTCGGCCCTGCAAGTTTCGTTGAGATAGGAATGGACCAAAAAGTTCCTCCTTTGTTTGAGAATGAGACAATCACGAAAATCGCAAAGGCTCATAACAAGACCCCGTCTCAAGTTTTGCTGCGTTGGGCCACTCAGAGAGGGATCGCCGTCATCCCAAAATCCAACAATGTTGAGCGGCAGACTCAAAACTTAGAATCTCTCGACTTTGACCTGACGGAGGCAGAAATTAAGGAAATATCAAATCTTAATAAGAATTTACGTTTCAACGATCCTGGAGTCTACGCAAATCTGCCTATATTTGCGTAAgttctgagaccgacc | Full length *Scheffersomyces stipitis Xyl*I gene, optimized |

*Uppercases illustrated amino acid sequence of target genes, while lowercases were overhangs for restriction enzymes


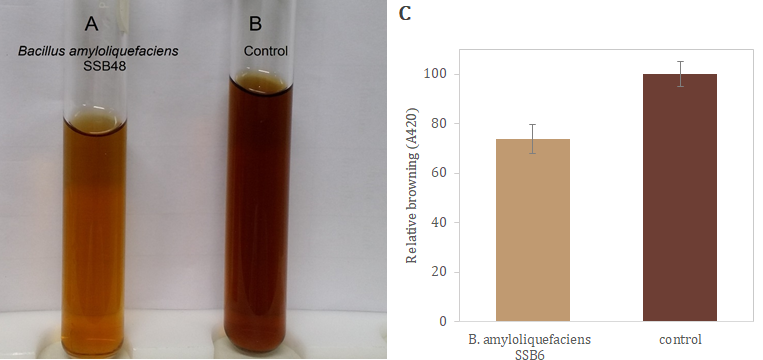


**Additional Figure S1**: Browning in crude-free supernatant of soy sauce broth. A = test tube containing media inoculated with *B. amyloliquefaciens strain SSB6* (initial inoculation of 6 log10CFU/ml.); B= test tube containing uninoculated media; C= bar chart of relative browning of B. amyloliquefaciens strain SSB6 versus uninoculated media (control) (100%) by measuring absorbance at 420nm. The incubation time was 14 days at room temperature (28-30°C) and all crude solid was removed. Samples prepared in duplicate, error bars represent ± SD.

**Additional Figure S2**: Xylose utilization and xylitol production of *Bacillus* strains A= pCG004-CbXR; B= pCG004-PkXR; C= pCG004-SsXR. Samples prepared in duplicate, error bars represent ± SD.

**Additional references**

Gilbert C, Howarth M, Harwood CR, Ellis T. Extracellular Self-Assembly of Functional and Tunable Protein Conjugates from Bacillus subtilis. ACS Synthetic Biology. 2017;6(6):957-67.

Lee ME, DeLoache WC, Cervantes B, Dueber JE. A Highly Characterized Yeast Toolkit for Modular, Multipart Assembly. ACS Synthetic Biology. 2015;4(9):975-86.
